# Buprofezin as a potent corrosion inhibitor for carbon steel in 1 M HCl solution

**DOI:** 10.1039/d5ra05962c

**Published:** 2025-12-17

**Authors:** Sally A. Abu Al-Khair, Rabab M. Abou-Shahba, Walaa A. Hussein, Ahmed A. El-Hossiany, Abd El-Aziz S. Fouda

**Affiliations:** a Department of Chemistry, Faculty of Science, Al-Azhar University Cairo Egypt; b Department of Chemistry, Faculty of Science, Mansoura University Mansoura-35516 Egypt asfouda@mans.edu.eg

## Abstract

Buprofezin was utilized to inhibit carbon steel (CS) corrosion in 1 M HCl, and corrosion behaviour was assessed using electrochemical (PDF, EIS) and chemical (ML) methods. Different techniques were used to identify the adsorbed film formed on the CS surface. Furthermore, the CS surface was examined using Fourier transform infrared spectroscopy (FTIR) to identify buprofezin bands. The experimental results indicate that buprofezin's efficiency increases with the inhibitor test solution concentration but decreases with increasing temperature. Using ML tests, buprofezin's inhibition efficiency (% IE) is 89.7% at 21 × 10^−6^ M, 25 °C and reached 81.3% at the same concentration and at 55 °C. According to the Langmuir adsorption isotherm, the buprofezin under investigation adsorbs on the CS surface. By computing a few pertinent thermodynamic parameters, the free energy of adsorption for the investigated buprofezin was established and discussed. Alongside the effectiveness of the inhibition, buprofezin's spontaneous adsorption on CS also increased. Polarization curves indicate that buprofezin is a mixed-type inhibitor that delays both cathodic hydrogen evolution and anodic CS dissolution. Theoretical studies confirmed the experimental results. The results show good agreement among all techniques.

## Abstract 2

Buprofezin was utilized to inhibit carbon steel (CS) corrosion in 1 M HCl using electrochemical analysis (potentiodynamic polarization, or PDP), chemical analysis (mass loss, or ML), and electrochemical impedance spectroscopy (EIS) testing.

## Graphical abstract

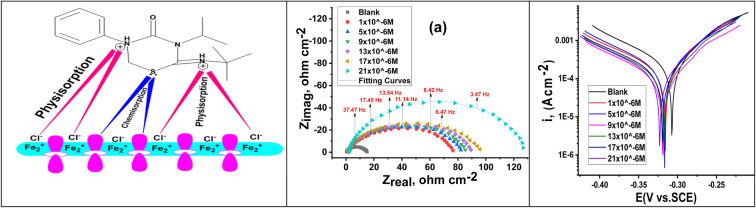

## 1. Introduction

Corrosion is a major industrial problem that damages many petroleum installations like reservoirs, distillation towers, and oil pipelines. While industries use methods such as galvanizing, electroplating, and inhibitors to mitigate these losses, using an inhibitor is considered the best method due to its simplicity and low cost.^1,2^ The use of acidic media to remove scale films and corrosion products is common in modified production processes. The pickling of metal alloys often requires the use of H_2_SO_4_ or HCl. Inhibitors are one of the greatest ways to stop metal reduction in acidic solutions .^3^ Organic compounds with nitrogen (N), oxygen (O), and sulfur (S) are often used as corrosion inhibitors because they can adsorb onto metal surfaces, blocking corrosive agents. Their effectiveness is often enhanced by polar functional groups, which improve their adsorption capabilities; these can be either organic or inorganic.^4^ The presence of lone pairs and π-electrons in inhibitor molecules allows electrons to move from the inhibitor to the metal surface and create a covalent bond. Natural and non-toxic substances are utilized to make these composites because they are environmentally acceptable.^5^ Stainless steel 304 was tested for corrosion resistance in molar HCl solutions using the following organic compounds: 4-(3-amino-5-(phenylamino)-1*H*-pyrazole-4-carboxamido)benzoic acid (B), 4-(2-cyano-3,3-bis(methylthio)acrylamido)benzoic acid (C), and 4-(3-phenyl-2-thioxo-2,3-dihydrothiazole-5-carboxamido)benzoic acid (A).^6^ Compounds A, B, and C showed inhibitory efficiency (% IE) of 96.8%, 86.1%, and 77.1%, respectively, according to the results. The novel chemical compound BFCPA (*N*-substituted-1,3-benzothiazol-2-pheny-2-[4-(furan-2-carbonyl)piperazin-1-phenyl]acetamide), which is derived from benzothiazole, was made independently. This novel compound was then employed as a corrosion inhibitor for mild steel in acidic solutions of 1 N and 2 N HCl.^7^ The system under investigation exhibited the highest inhibitory efficiencies of 89.32% in 1 N HCl and 82.71% in 2 N HCl when maintained at room temperature for two hours with an optimal concentration of 100 ppm.

*N*-(Butylidene)-5-(3,3-dimethyltriaz-1-en-1-yl)-1*H*-imidazole-4-carboxamide (BDIC)^8^ was used to inhibit carbon steel from corroding in a solution of 1 M HCl. The higher the concentration of BDIC, the better it was at preventing corrosion. At 25 °C and a BDIC concentration of 1 × 10^−3^ M, the highest inhibitory efficiencies were 90.2% determined by electrochemical impedance spectroscopy (EIS), 89.5% by weight loss (WL), and 90.2% determined by potentiodynamic polarization (PDP). To inhibit CS from corroding, a novel hydrazone chemical called *N*'-[(*Z*)-(4-chlorophenyl)methylidene]-2-(5-methoxy-2-methyl-1*H*-indol-3-yl)acetohydrazide (HTH) was used in a 1.0 mol L^−1^ HCl solution.^9^ Carbon steel was 89% protected by hydrazone in the 303–333 K temperature range, showing high resistance to temperature effects. The ability of two newly synthesized triazole derivatives, 3,5-di(*m*-tolyl)-4*H*-1,2,4-triazole (*m*-THT) and 5-di(*m*-tolyl)-4-amino-1,2,4-triazole (*m*-DTAT), to inhibit corrosion in a 1 M HCl solution was investigated by El Mehdi and colleagues.^10^ In this acid, both compounds showed outstanding mild steel corrosion inhibition performance; however, *m*-DTAT outperformed *m*-THT with an efficiency of 95% compared to 91%. In another study, Zhang *et al.*^11^ investigated how a newly developed oxadiazol triazole derivative for mild steel inhibited corrosion in sulfuric acid. Their results showed that, at 298 K, this chemical had an efficiency of over 97.6%, making it an efficient corrosion inhibitor. The corrosion behaviour of mild steel in 0.1 M HCl solution without and with 5-amino-1,2,4-triazole (5-ATA), 5-amino-3-mercapto-1,2,4-triazole (5-AMT), 5-amino-3-methylthio-1,2,4-triazole (5-AMeTT) or 1-amino-3-methylthio-1,2,4-triazole (1-AMeTT) was studied.^12^ Increasing temperature was found to greatly enhance IE% till reaching a plateau at about 80% for 5-ATA and more than 90% for the other compounds between 323 and 348 K. Two triazole derivatives, namely 1-[2-(4-nitro-phenyl)-5 [1,2,4]triazol-1-ylmethyl-[1,3,4]oxadiazol-3-yl]-ethanone (NTOE) and 1-(4-methoxy-phenyl)-2-(5-[1,2,4]triazol-1-ylmethyl-4H[1,2,4]triazol-3-ylsulfanyl)-ethanone (MTTE), were tested as corrosion inhibitors for mild steel in 1 M hydrochloric acid solutions.^13^ The inhibition efficiency increased with inhibitor concentration, reaching a maximum value of 99.3% for NTOE and 98.8% for MTTE at 10^−3^ M, 25 °C. Two Schiff bases, 2-{[(4-methoxyphenyl)imino]methyl}phenol (SB-1) and 1-{[(4-methoxyphenyl)imino]methyl}-2-naphthol (SB-2), were studied in 0.1 M and 1 M H_2_SO_4_ as corrosion inhibitors for steel by Hasanov *et al.*^14^ The inhibition efficiency in 0.1 M H_2_SO_4_ is 84% for SB-1 and 93% for SB-2 at 20 mM, 25 °C, but 78% for SB-1 and 88% for SB-2 in 1 M H_2_SO_4_ at the same concentration and temperature.

Buprofezin is an insecticide that acts as a chitin synthesis inhibitor, specifically targeting sucking pests such as whiteflies, plant hoppers, and scale insects. It kills insects, particularly nymphs, by interfering with the formation of their shells, which prevents them from molting. Buprofezin has low toxicity to humans and other mammals and is used in a range of agricultural settings, including on fruit and vegetable crops, as well as in public health pest control.

Despite its primary function as an insecticide, buprofezin may also have corrosion-inhibiting properties. Although not being its main commercial application, its molecular structure—particularly the presence of a ring containing sulfur—indicates that it may interact with metal surfaces to create protective coatings that prevent corrosion. The possible applications of buprofezin as a corrosion inhibitor include: (i) preventing the corrosion of metal parts in agricultural equipment in humid conditions; (ii) it treats a variety of insect pests, particularly in fruit and vegetable crops; (iii) preventing corrosion in pipelines or storage tanks where contamination from buprofezin may already be present. By altering the structure of buprofezin, it is possible to facilitate the development of novel corrosion inhibitors. Buprofezin was chosen as a corrosion inhibitor because: (i) O, N, and S atoms are active sites in the buprofezin molecule; (ii) this substance appears to be environmentally friendly and significant for biological study,^15^ and (iii) buprofezin is easy to prepare and purify.^16^ By using modified tests, buprofezin was evaluated as a corrosion inhibitor for high carbon steel in acidic environments and showed a high percentage of IE.

The use of buprofezin as a corrosion inhibitor for CS in acidic environments is examined in this study using a variety of techniques. In addition to discussing and calculating thermodynamic parameters, various methods were used to examine its surface morphology.

## 2. Experimental tests

### 2.1. Material preparation

Carbon steel coins with the following chemical composition (in weight percentage) were prepared: 0.18% C, ≤0.05% S, 0.60–0.90% Mn, ≤0.04% P, and the remainder as iron. These 20 mm long, 20 mm wide, and 2 mm thick coins were abraded using 400–1200 grade emery sheets and degreased with acetone. After being scrubbed with an abrasive cleaner, they were allowed to air dry at room temperature.

### 2.2. Test solutions

**Fig. 1:**
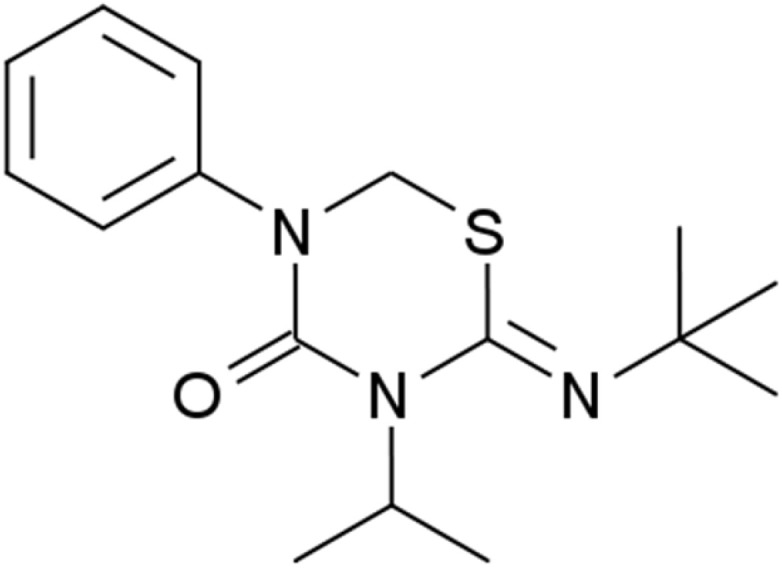
Buprofezin: (*Z*)-2-*tert*-butylimino-3-isopropyl-5-phenyl-1,3,5-thiadiazinane-4-one. Chemical formula: C_16_H_23_N_3_OS. Molecular weight: 305.44.

Analytical reagent grade 37% HCl was diluted with bi-distilled water to create the aggressive solution used in this study. A standard stock solution of buprofezin with a concentration of 10^−3^ M was prepared by dissolving 0.0305 g of the compound in 10 mL of dimethyl formamide. The volume was then made up to 100 mL with ethanol. To obtain the desired concentrations, this stock solution was diluted with bi-distilled water. A buprofezin concentration range of 21 × 10^−6^ M to 1 × 10^−6^ M was used. The structure of buprofezin is shown in Fig. 1.

### 2.3. Non-electrochemical tests

#### 2.3.1 ML tests

Before measurement, the samples were prepared by abrading with abrasive paper, washing with bi-distilled water, and degreasing with acetone. They were then dried in a flow of air. The ML tests were conducted in a 100 mL beaker, with the samples immersed in 100 mL of a deaerated solution. The samples were held upright in the solution using glass hooks, and the experiments were performed under static conditions. Seven experiments were conducted in different environments: a control and six others with varying concentrations of inhibitor (1 × 10^−6^, 5 × 10^−6^, 9 × 10^−6^, 13 × 10^−6^, 17 × 10^−6^, and 21 × 10^−6^ M) in HCl. After three hours, the materials were removed, rinsed with bi-distilled water and acetone, dried, and reweighed. The samples were initially weighed with a precision of ±0.0001 g before placing them in the electrolyte. The corrosion rate (*k*_corr_) of the metal samples was determined using the following relation:^17^

1 *k*_corr_ = Δ*w*/*AT*

where, Δ*w* (mg) is the reduction in mass, *A* (cm^2^) is the area of the surface of CS and *T* (min) is the time. The % IE and the surface coating (*θ*) of buprofezin were computed according to the following equations:^18^

2 % IE = [1 − (CR_inh_/CR_free_)] × 100

3 *θ* = [1 − CR_inh_/CR_free_]

where CR_free_ and CR_inh_ are the corrosion rates without and with buprofezin, respectively. The ML measurements and the calculation of corrosion rates are carried out according to ASTM G1 and ASTM G31.

### 2.4. Electrochemical tests

An electrochemical test setup consisting of three electrodes was used, assembled in a double Pyrex glass cell. The working electrode (WE) is CS, the reference electrode is a fine Luggin capillary linked to a saturated calomel electrode (SCE), and the auxiliary electrode is platinum foil. The WE was created from CS square coins, each of which has an active surface area of 1 cm^2^ on one side. The electrode surface was treated in the same way as for ML testing. Every experiment was run at 25 °C using fresh unstirred medium.

#### 2.4.1 PDP technique

Tafel polarization curves were generated at 25 °C, covering a potential range of −250 to 250 mV/SCE at a slow scanning speed of 0.2 mV s^−1^, which ensured quasi-stationary conditions. Before plotting these curves, the electrode was held at its zero potential for 30 minutes to achieve a steady-state potential. Log *i*_corr_ and *E*_corr_ for HCl alone and in the presence of buprofezin were obtained by extrapolating the cathodic and anodic Tafel lines. Then *i*_corr_ was utilized to compute the % IE and (*θ*) using eqn (4):^19^4
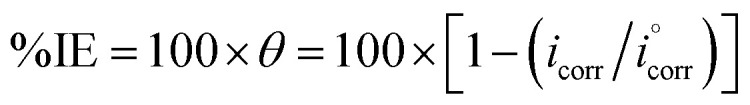
where *i*_corr(inh)_ and *i*_corr(free)_ are the currents obtained from corrosion in the presence and absence of buprofezin, respectively. From the plot, the correlation between the potential (*E*_corr_) and the logarithm of the corrosion current (log *i*_corr_) was determined. The corrosion potential (*E*_corr_) and the Tafel slopes (*β*_a_ and *β*_c_) were also predicted from this plot.

#### 2.4.2 EIS technique

Electrochemical impedance spectroscopy (EIS) measurements were conducted at an open-circuit potential, with a signal amplitude of 10 mV and frequency range of 100 kHz to 10 Hz. From the Nyquist diagrams, one can measure *R*_ct_ and *C*_dl_. The % IE and *θ* were calculated utilizing eqn (5):^20^5
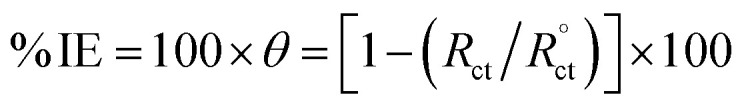
where *R*^0^_ct_, *R*_ct_ are the resistances of the uninhibited and inhibited buprofezin, respectively.

All the electrochemical corrosion methods were carried out after 30 min immersion period for CS to achieve a steady open-circuit potential (OCP). *R*_ct_ (charge transfer resistance) and *C*_dl_, (double-layer capacitance) are the parameters obtained from EIS. Using Gamry apparatus (PCI4/750), measurements were conducted using computer framework software DC 105 for PDP testing and EIS 300 for EIS. Gamry categorization was based on the ESA 400. Gamry Echem Analysis 5.5 was utilized for data analysis, fitting, and simulations. The standard deviation does not exceed 5%.

### 2.5. Surface characterization

The samples were immersed in buprofezin and blank solutions for a whole day. Following their removal and drying, the specimens were analyzed using a range of techniques to determine the chemical composition and characteristics of the resulting surface layer.

#### 2.5.1 Scanning electron microscopy (SEM) tests

The surface morphology of the specimens before and after corrosion was analyzed using JEOL JSM-5500.

#### 2.5.2 Energy dispersive X-ray (EDX) tests

EDX (energy dispersive X-ray spectroscopy) was used to identify the components on the surface.^21^

#### 2.5.3 Atomic force microscope (AFM) tests

AFM gives the morphological properties of the CS metal surface. This test is conducted in 1 M HCl without a buprofezin inhibitor and at the greatest possible concentration of buprofezin (21 × 10^−6^ M). After cleaning it with double-distilled water and allowing it to dry, its surface properties were evaluated.^22^ Using the Park systems, XE-100 model, AFM was carried out in contact mode.

#### 2.5.4 FTIR tests

FTIR analysis was carried out using an IR (PerkinElmer) spectrophotometer at the central lab, Faculty of Pharmacy, Mansoura University, Egypt, to analyze the CS surface alone and in the presence of a coated film from buprofezin in order to identify the presence of functional groups characteristic of buprofezin after adding a concentration of 21 × 10^−6^ M in a solution of 1.0 M HCl without dipping the CS metal coins, and with the same concentration in 1.0 M HCl after dipping the CS for three hours.

**Fig. 2:**
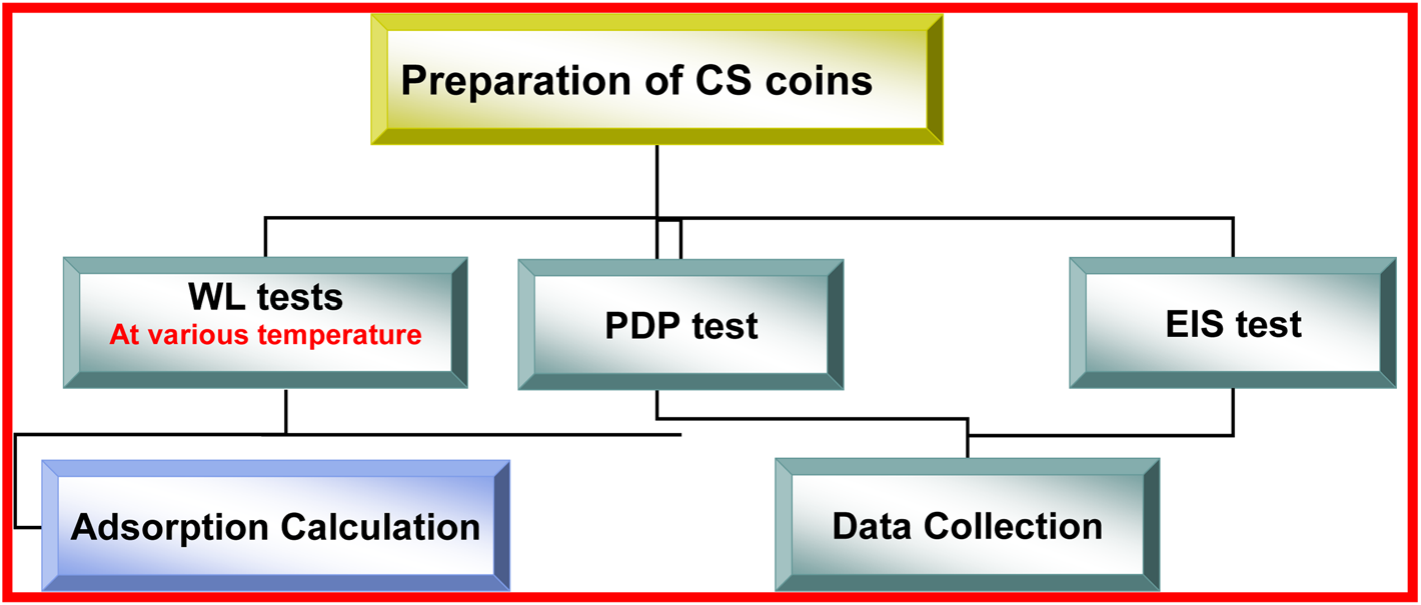
Flowchart summarizing the methodology in the corrosion inhibition study.

The flowchart in Fig. 2 provides a visual representation of the systematic approach employed in this study to assess the corrosion inhibition performance of buprofezin on CS in a 1.0 M HCl solution.

## 3. Results and discussion

### 3.1. ML tests

**Fig. 3:**
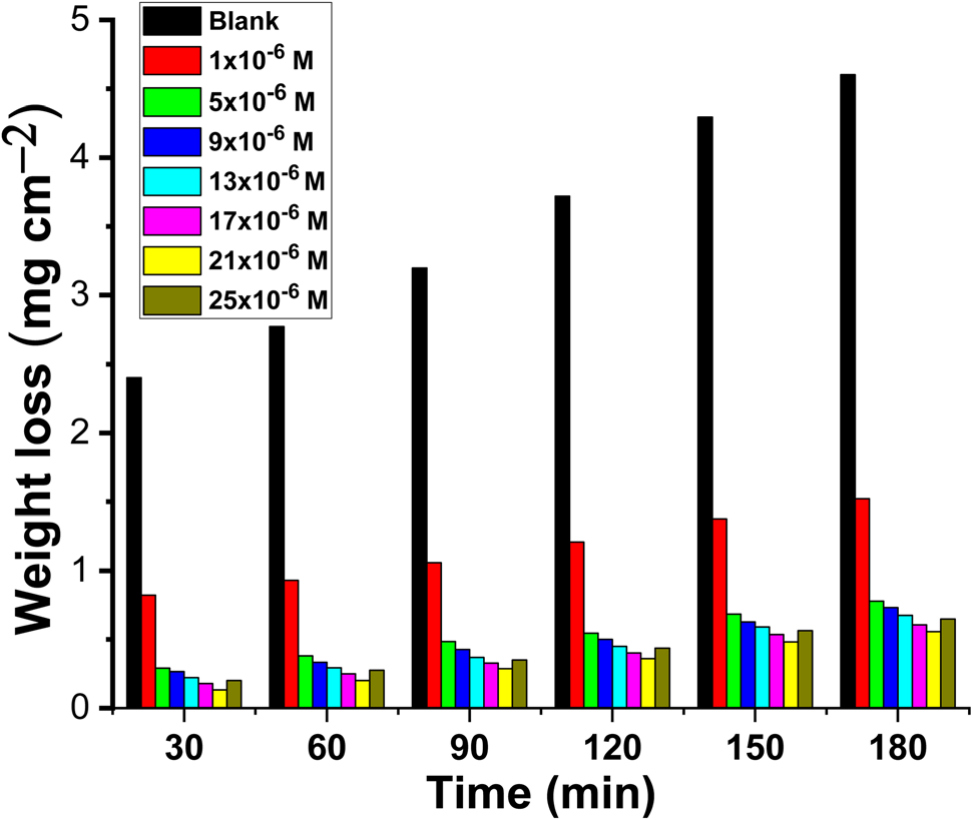
Time-mass-loss plots for CS dissolution in 1.0 M HCl with and without different concentrations of buprofezin at 25 °C.

ML was performed on CS in 1.0 M HCl with and without varying doses of buprofezin; the outcomes are displayed in Fig. 3. Table 1 contains the calculated percentage IE, *k*_corr_, and *θ* values. It is evident that the percentage IE rises with buprofezin concentration and decreases with temperature (from 25 to 55 °C). The enhanced corrosion inhibition, which effectively inhibited corrosion, was brought about by the addition of heteroatoms on the CS surface. The results indicated that buprofezin has good anti-corrosive characteristics, which is in line with recent studies on heterocyclic compounds.^23–26^ With increasing buprofezin concentration, *k*_corr_ decreased while the inhibition efficiency (IE) increased.^27^ The addition of a concentration of 21 × 10^−6^ M produced the highest inhibitory efficiency, which subsequently dropped at a concentration of 25 × 10^−6^ M, most likely as a result of the physical adsorption mechanism. The solution reaches saturation when the inhibitor concentration is greater than 21 × 10^−6^ M, which is the ideal concentration. Additionally, a significant interaction between the molecular inhibitor in the solution and the inhibitor affixed to the metal surface may occur. The inhibitor's coating may be released into the solution as a result of this potential.

**Table 1:** Impact of temperature on *k*_corr_, *θ*, and % IE of MS with and without different concentrations of buprofezin

Temp., °C	[Buprofezin], M	*k*_corr_ (mg cm^−2^ min^−1^) ±0.0002–0.0006	*θ*	% IE
25	Blank	0.0310	—	—
	1 × 10^−6^	0.0101	0.675	67.5
	5 × 10^−6^	0.0041	0.867	86.7
	9 × 10^−6^	0.0035	0.886	88.6
	13 × 10^−6^	0.0035	0.886	88.6
	17 × 10^−6^	0.0033	0.892	89.2
	21 × 10^−6^	0.0030	0.903	90.3
	25 × 10^−6^	0.0037	0.882	88.2
35	Blank	0.0560	—	—
	1 × 10^−6^	0.0254	0.546	54.6
	5 × 10^−6^	0.0129	0.769	76.9
	9 × 10^−6^	0.0100	0.821	82.1
	13 × 10^−6^	0.0085	0.848	84.8
	17 × 10^−6^	0.0069	0.877	87.7
	21 × 10^−6^	0.0058	0.897	89.7
45	Blank	0.1019	—	—
	1 × 10^−6^	0.0603	0.408	40.8
	5 × 10^−6^	0.0322	0.684	68.4
	9 × 10^−6^	0.0229	0.775	77.5
	13 × 10^−6^	0.0195	0.809	80.9
	17 × 10^−6^	0.0148	0.855	85.5
	21 × 10^−6^	0.0133	0.869	86.9
55	Blank	0.2110	—	—
	1 × 10^−6^	0.1437	0.319	31.9
	5 × 10^−6^	0.0831	0.606	60.6
	9 × 10^−6^	0.0627	0.703	70.3
	13 × 10^−6^	0.0525	0.751	75.1
	17 × 10^−6^	0.0454	0.785	78.5
	21 × 10^−6^	0.0395	0.813	81.3

### 3.2. Effect of temperature and kinetic–thermodynamic parameters

**Fig. 4:**
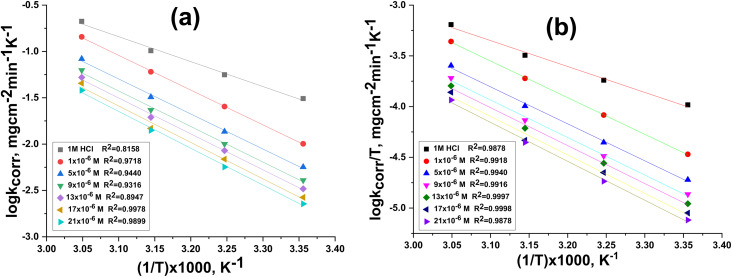
(a) Log* k*_corr_ and (b) log* k*_corr_/*T vs.* 1/*T* curves for CS with and without different concentrations of buprofezin.

Temperature can alter how a chemical behaves in a corrosive environment and change the interaction between steel and an inhibitor. As the temperature rises, the inhibitor's inhibition efficiency (IE) slightly decreases. The decrease in the % IE as the solution temperature rises may be due to the movement of the inhibitor molecules, which in turn reduces the interaction between the inhibitor molecules and the CS surface. This suggests that the inhibitor molecules are adsorbed physically on the CS surface. The energy of activation (E*) for dissolution of the CS was measured from the slope of log* k*_corr_ against 1/*T* by applying the Arrhenius equation (eqn (6)) (Fig. 4a):^28^6
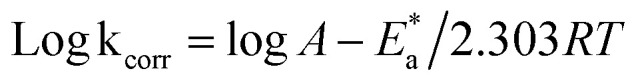
where *A* is the Arrhenius pre-exponential element. 
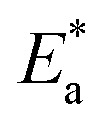
 is increased in the existence of the buprofezin than in its absence. The influence of the temperature on IE and *k*_corr_ is presented in Table 2. There is a rise in the activation energy with increasing concentration of buprofezin, which led to the rise of the thickness of the barrier layer designed on the CS surface. This increase is due to the adsorption nature of buprofezin on the CS surface and corresponds to the physical adsorption. From thetansitional state equation, the changes in entropy and enthalpy were calculated.^29^7
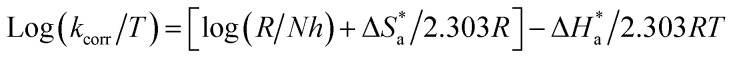
where (*h*) is Planck's constant. Fig. 4b shows straight lines resulting from the plot of log(*k*_corr_/*T*) against 1000/*T*, which shows the transitional state of buprofezin. Slopes given from the curves of Fig. 4b were utilized to measure the enthalpy 
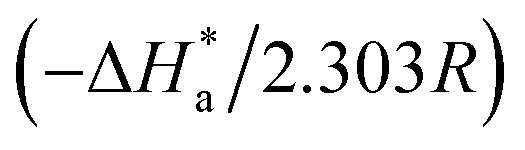
, and the activation entropy was measured utilizing the intercept of these lines 
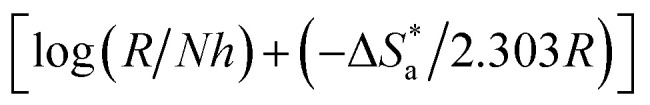
. The negative values of ΔH* indicate that the formation of the activated complex is an exothermic process. A negative entropy of activation (ΔS*) indicates that the activated complex is more ordered than the reactants, reflecting a decrease in entropy as the reactants transform into the activated complex.^30,31^ These results also support the established thermodynamic relationship between the apparent activation energy 
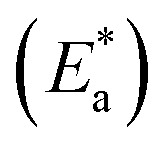
 and the enthalpy of activation (ΔH*) for unimolecular reactions.^32^8
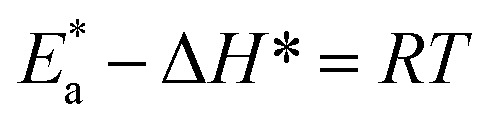


**Table 2:** Kinetic parameters of activation as a function of inhibitor concentration

Conc. M	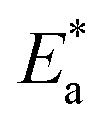 , kJ mol^−1^	−ΔH*, kJ mol^−1^	−ΔS*, J mol^−1^K^−1^
Blank	51.5	48.9	109
1 × 10^−6^	71.7	69.1	51
5 × 10^−6^	72.3	69.7	53
9 × 10^−6^	73.4	70.8	53
13 × 10^−6^	74.1	71.5	52
17 × 10^−6^	75.1	72.5	50
21 × 10^−6^	76.1	73.4	49

As the calculated value (2.6 at 25 °C) aligns closely with those estimated in Table 2, it suggests that the inhibitor consistently affects both 
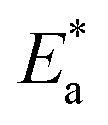
 and ΔH*.

### 3.3. Adsorption isotherms

Numerous isotherms were utilized to fit the data,^33^ but the best fit was obtained with the Langmuir adsorption isotherm with a linear correlation coefficient (*R*^2^ > 0.9975) close to 1, and all slope values are close to unity.^34^ Langmuir isotherm can be obtained from the next equation:^35^

**Fig. 5:**
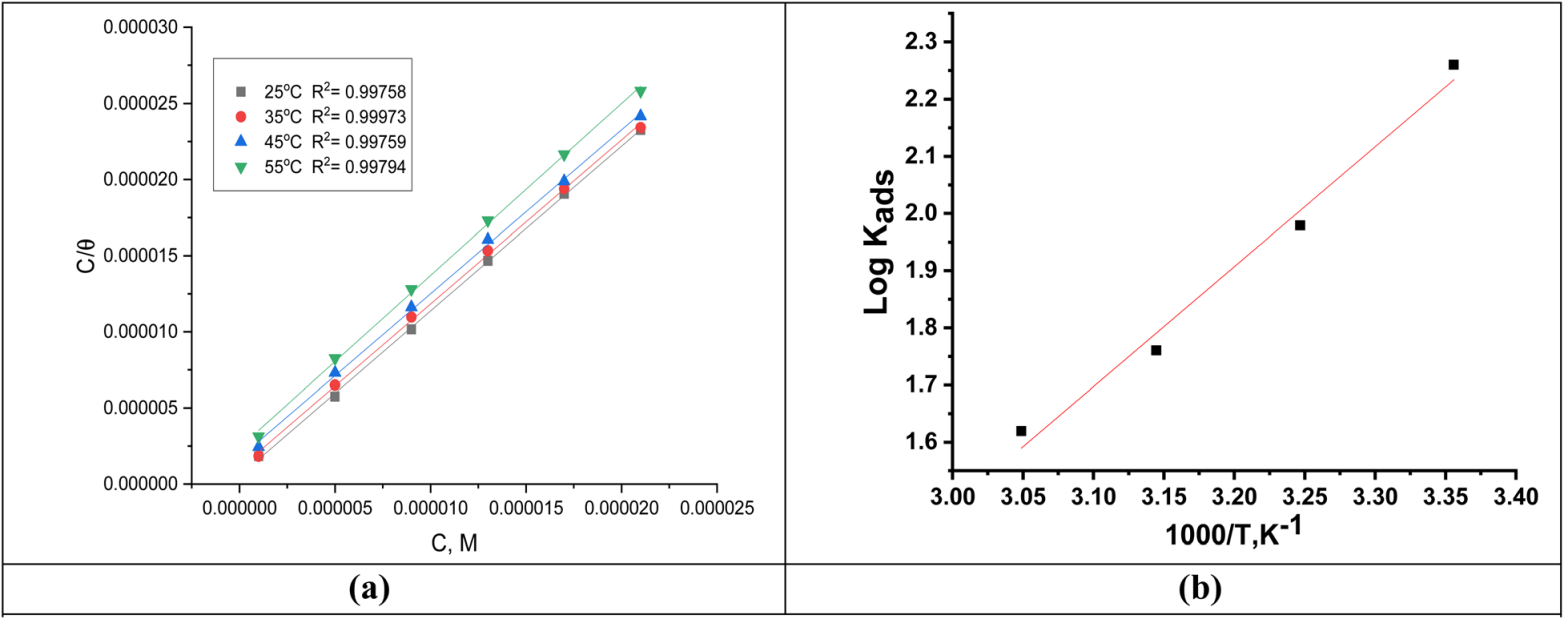
(a) Langmuir plots for CS in 1 M HCl and in the presence of different concentrations of buprofezin at different temperatures. (b) Plots of log *K*_ads_*vs.* 1/*T* for the adsorption of buprofezin in 1 M HCl.

9 *C*/*θ* = 1/*K* + *C*

where *C* is the concentration of buprofezin and *K*_ads_ is the adsorption equilibrium constant. The adsorption equilibrium constant expressed as *K*_ads_ can be calculated from Fig. 5a, the difference among *C/θ* and *C* where *θ* is the surface coverage, = IE/100. The 
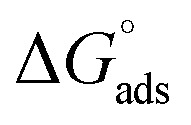
 and *K*_ads_ data are reported in Table 3. The 
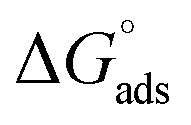
 is calculated using eqn (10):^36^10
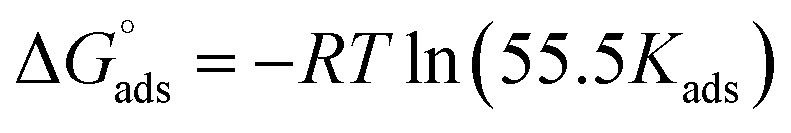
In this case, *T* is the absolute temperature, *R* is the universal gas constant, and 55.5 is the water concentration in mol L^−1^. The strength of the double layer adsorbed on the metal surface, and the spontaneity of the process are due to the higher values of *K*_ads_ and the negative values of 
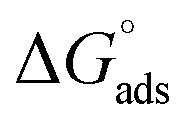
.^37^ The literature^38^ states that physical adsorption, which is defined by a charged molecule/charged metal contact, is indicated by 
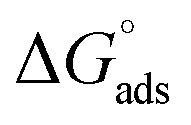
 values of −20 kJ mol^−1^ or above. Conversely, chemisorption, which involves the transfer of charge between organic molecules and a metallic substrate, is indicated by values that are close to −40 kJ mol^−1^ or more negative. A mixed adsorption mechanism comprising both physical (physisorption) and chemical (chemisorption) processes is indicated by the 
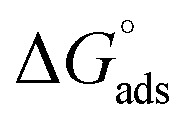
 values of 22.8–21.1 kJ mol^−1^.^39^ The van't Hoff equation can be used to measure 
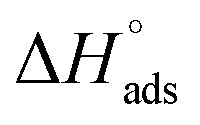
 (Fig. 5b) and 
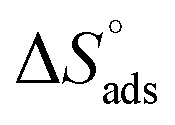
 expressed by:11
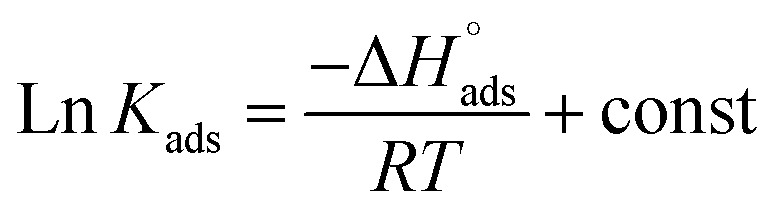


**Table 3:** Langmuir data for CS without and with varying buprofezin contents at (30–50 °C)

Temp., K	*K*_ads_ × 10^3^ M^−1^	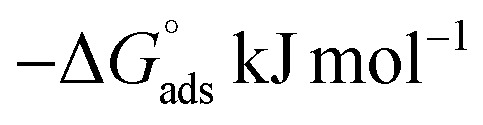	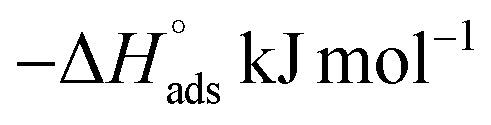	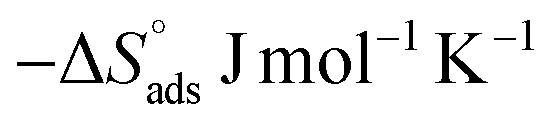
298	181	22.8	40.0	76.5
308	95	21.9		71.1
318	57	21.3		66.9
328	41	21.1		64.2

The entropy 
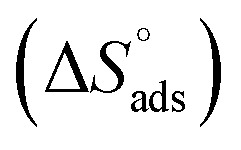
 and standard enthalpy 
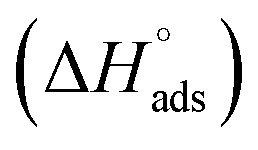
 of adsorption were determined by applying eqn (8). As seen in Fig. 5b, these numbers were computed using the plot's slope and intercept, respectively. The small and negative 
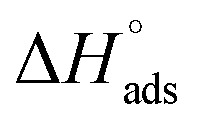
 values show that buprofezin adsorption is an exothermic process that is compatible with physical adsorption. Additionally, when buprofezin is administered, the 
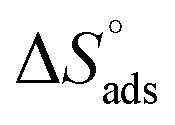
 sign is negative, indicating spontaneous adsorption and a reduction in the disorder related to the corrosion process (Table 3).

### 3.4. Measurements of OCP

**Fig. 6:**
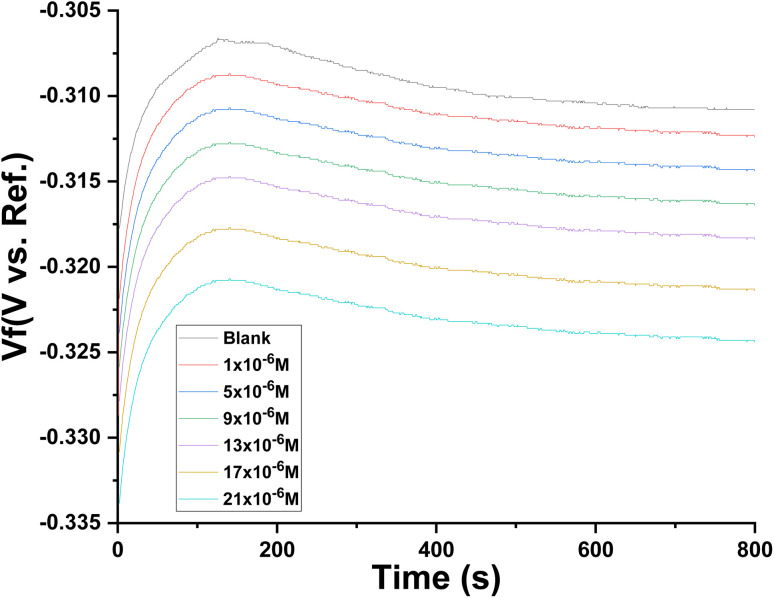
*E*_OCP_ *vs.* time curves for CS in the 1.0 M HCl alone and with various concentrations of buprofezin at 25 °C.

Fig. 6 displays the relation of the OCP *vs.* time curves for CS in 1.0 M HCl in the absence and presence of different concentrations of the investigated compound (buprofezin) at 298 K. As the deterioration of the CS occurred as the protective oxide film on its surface dissolved in the corrosive environment. From the OCP curves, it is noted that the potentials of inhibited solutions moved to more negative values compared to the uninhibited.

### 3.5. PDP tests

**Fig. 7:**
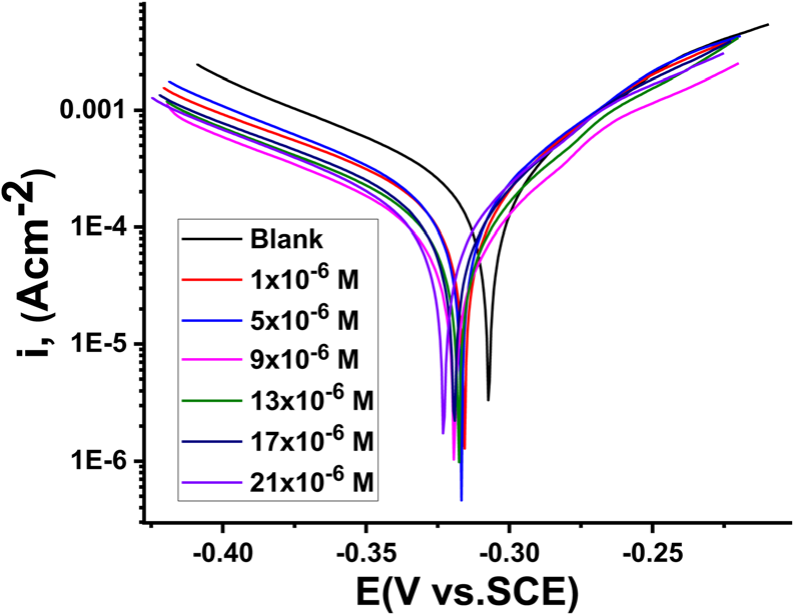
PDP curves for the CS dissolution with and without different concentrations of buprofezin at 25 °C.

The PDP technique was used to create a coating that inhibited corrosion on the surface of CS.^40^ Polarization curves for CS at various concentrations of buprofezin in aerated 1 M HCl solutions are shown in Fig. 7.

It can be seen from Fig. 7 that the introduction of buprofezin into 1 M HCl shifts *E*_corr_ in the cathodic direction and inhibits the anodic and cathodic processes. This indicates that buprofezin predominantly inhibits the corrosion mechanism by controlling the anodic reactions. Additionally, the cathodic sites of the metal surface shift to negative potentials, indicating that buprofezin can also act as a cathodic inhibitor, with the inhibitor molecules adsorbing on the cathodic sites, and thereby suppressing the cathodic reactions. From Fig. 7, both the cathodic and anodic reactions are inhibited, and the inhibition increases with the increase in inhibitor concentration in solution. The extrapolation of the Tafel straight line enables the calculation of the corrosion current density (*i*_corr_). Table 4 displays the corrosion properties, including the Tafel slopes (*β*_a_, *β*_c_), inhibition efficiency (% IE), corrosion potential (*E*_corr_), surface coverage (*θ*), corrosion rate (*k*_corr_), and corrosion current density (*i*_corr_). The change in *β*_a_ and *β*_c_ values, as shown in Table 4, indicates that adsorption of buprofezin modifies the mechanism of both corrosion partial reactions and cathodic hydrogen evolution.^41^ The decrease in corrosion current density (*i*_corr_) demonstrated that the adsorption of buprofezin can reduce the corrosion rate of CS. The results in Table 4 show that there is no definite trend in the shift of *E*_corr_ values in the presence of various concentrations of buprofezin in 1 M HCl solution.^42^ This result indicates that buprofezin can be classified as a mixed-type inhibitor in 1 M HCl solution. It is seen that inhibition efficiency increases with increasing buprofezin concentration due to more inhibitor molecules being adsorbed on the surface of the CS, which consequently diminishes the solubility of the surface layer^43^ The inhibition efficiency (% IE) reached its highest value of 88.5% at a concentration of 21 × 10^−6^ M. The results are in very good agreement with those obtained from weight loss measurements.

**Table 4:** PDP results for CS dissolution with and without different concentrations of buprofezin at 25 °C

Conc., M	*i*_corr_, µA cm^−2^	−*E*_corr_, mV *vs.* SCE	*β*_a_, mV dec^−1^	−*β*_c_, mV dec^−1^	*k*_corr_ mg cm^−2^ min^−1^	*θ*	%IE
HCl	940	307	70	148	0.0747	—	—
1 × 10^−6^	281	315	72	161	0.0633	0.701	70.1
5 × 10^−6^	250	316	80	174	0.0549	0.734	73.4
9 × 10^−6^	243	319	89	173	0.0531	0.741	74.1
13 × 10^−6^	242	317	80	151	0.0525	0.742	74.2
17 × 10^−6^	154	319	87	178	0.0404	0.836	83.6
21 × 10^−6^	108	322	88	205	0.0303	0.885	88.5

### 3.6. EIS tests

**Fig. 8:**
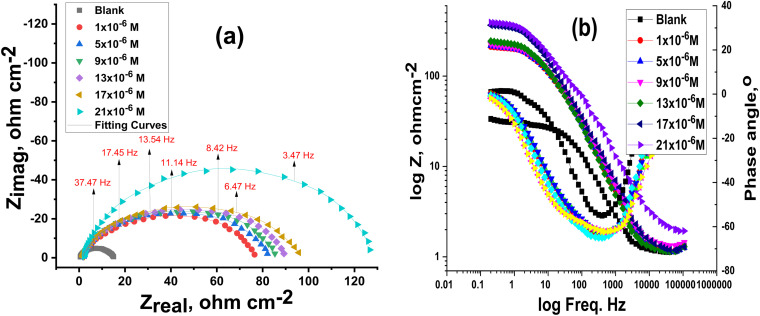
Nyquist (a) and Bode (b) diagrams for CS dissolution with and without different concentrations of buprofezin at 25 °C.

**Fig. 9:**
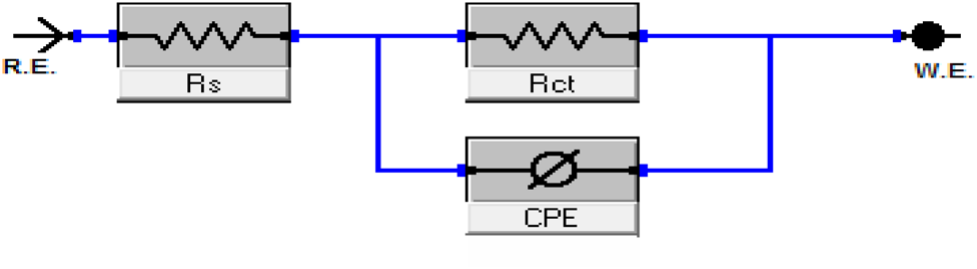
Electrical circuit utilized to fit the EIS data.

EIS was used to examine the electrode/electrolyte interaction and corrosion processes on the CS surface, both with and without buprofezin. EIS measurements were conducted at 25 °C and at open-circuit potential (OCP) over a wide frequency range to fully characterize these phenomena. Fig. 8a and b displays the Nyquist and Bode curves generated at OCP at 25 °C with varying doses of buprofezin, respectively. When buprofezin was absent, both the Nyquist and Bode plots showed a single arc, which corresponds to a single time constant. The capacitive loop diameters increase in the presence of buprofezin, indicating an improvement in the thickness of the adsorbed layer on the CS surface. Fig. 8a illustrates how the impedance spectra show a single, imperfect capacitive loop. The interfacial impedance frequency disperses due to the heterogeneity of the electrode surface, producing this defect.^44,45^ This heterogeneity is not just a natural feature of the electrode surface but can also be brought on by contaminants, roughness, adsorption, and dislocations.^46^ This type of spectrum typically shows that a charge transfer activity is occurring on a heterogeneous and uneven substrate.^47^ To calculate the EIS data for buprofezin, the excellent equivalent circuit in Fig. 9 was utilized; it fits the results well and exhibits a clear charge transfer. The CPE is put in the circuit in place of a pure double-layer capacitor to obtain a maximum precise fit.^48,49^*C*_dl_ was determined utilizing eqn (12) and (13):

12 *C*_dl_ = *Y*_0_(*ω*_max_)^*n*−1^

13 *ω*_max_ = 2π*f*_max_

where *Y*_0_ = the CPE degree and *f*_max_ is the frequency at which the imaginary constituent of the EIS is highest. The obtained plots are very parallel for all samples with and without altered concentrations of buprofezin, showing that there is no change in the mechanism of corrosion.^50^ According to the data in Table 5, the percentage of IE increased because the *R*_ct_ data increased as the buprofezin concentration increased. *R*_ct_ increases with inhibitor concentration due to the formation of an adsorbed protective layer that hinders the corrosion (charge transfer) process. According to the data in Table 5, adding buprofezin significantly lowers the value of *Y*_0_ compared to the bulk solution. The fact that the *Y*_0_ for the blank solution is higher than that of the inhibited solution suggests that the buprofezin molecules interact with the electrode surface, which in turn minimizes corrosion on the electrode's exposed areas. The data also show that adding buprofezin decreases the values of double-layer capacitance (*C*_dl_), likely due to a reduction in the local dielectric constant and/or an increase in the thickness of the electric double layer. This further supports the conclusion that buprofezin works by adsorbing onto the carbon steel (CS)/solution interface.^51,52^ The increase in *R*_s_ with inhibitor concentration is mainly because the inhibitor modifies the ionic conductivity of the electrolyte (increasing viscosity or reducing ion mobility). The values of the exponent ‘*n*’ typically range from 0 to 1, and are influenced by factors like electrode surface roughness, heterogeneity, and dielectric constant.^53,54^ In our investigation, the value of ‘*n*’ for 1 M HCl alone was higher than when it was absent. This indicates surface irregularities caused by the corrosion-induced roughening of the CS. The fitted results aligned well with the experimental data, as evidenced by the small chi-squared values (Table 5). The double-layer capacitance (*C*_dl_) was calculated using eqn (10),^55,56^ and the results showed good agreement with measurements obtained from all techniques. It is also interesting that the results obtained from the EIS data showed good agreement with the results obtained from WL and PDP measurements.

**Table 5:** EIS data for CS dissolution without and with different concentrations of buprofezin at 25 °C

Conc., M	*R*_s_, Ω cm^2^	*Y*_0_, µ Ω^−1^ s^*n*^ cm^−2^ × 10^−6^	*n*	*R*_ct_, Ω cm^2^	*C*_dl_, ×10^−6^, µF cm^−2^	*θ*	% IE	Goodness of fit (*χ*^2^)
1 M HCl	1.001	744	0.854	11	701	—	—	22.14 × 10^−3^
1 × 10^−6^	1.392	499	0.792	77	461	0.857	85.7	19.13 × 10^−3^
5 × 10^−6^	1.419	522	0.792	82	482	0.866	86.6	17.97 × 10^−3^
9 × 10^−6^	1.424	510	0.770	84	485	0.869	86.9	20.74 × 10^−3^
13 × 10^−6^	1.533	495	0.759	85	460	0.871	87.1	21.78 × 10^−3^
17 × 10^−6^	1.657	464	0.740	95	436	0.884	88.4	17.67 × 10^−3^
21 × 10^−6^	1.997	348	0.726	126	298	0.913	91.3	18.57 × 10^−3^

### 3.7. Surface examination study

#### 3.7.1 Fourier transform infrared spectroscopy (FTIR) analysis

**Fig. 10:**
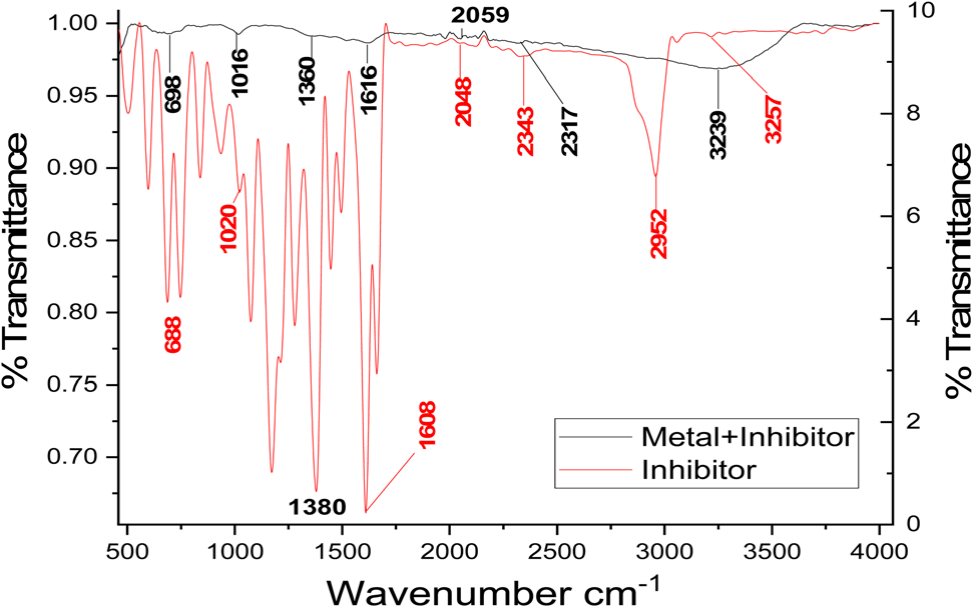
FTIR spectra of pure buprofezin and of the film found on the CS surface after immersing in a solution containing buprofezin.

FTIR spectrophotometry is a proven and effective method for determining the nature of bonds that exist between particular functional groups in organic molecules. The coating film that formed on the surface of carbon steel (CS) was investigated in this work by means of the FTIR spectrum.^57^Fig. 10 displays the FTIR band of pure buprofezin, which possesses an active band at 1280 cm^−1^ because of the –C–F stretching frequency of the alkyl halide group. Both organic and inorganic materials can be detected using ATR-FTIR using both quantitative and qualitative investigation. To determine a molecule's chemical bonds, an infrared absorption spectrum was produced. Functional groups have been found, and covalent bonding information has been described using FTIR, a crucial analytical method. The corrosion product of CS is not IR active, as the ATRFT-IR spectrum of the corrosion product at the CS surface in 1 M HCl does not display any helpful adsorption peaks. The fingerprint spectra of the stock buprofezin and the CS surface were obtained after a 24-hour immersion in 1 M HCl and 21 × 10^−6^ M of buprofezin. When compared to one another, it was evident that the CS surface had the same fingerprint as the stock buprofezin solution, with the exception of the absence of a functional group, which was thought to be caused by a reaction with HCl. The peaks at the CS surface in Fig. 10 show a slight shift from the stock inhibitor solution's initial peak; this movement suggests that CS and part of the inhibitor molecules are interacting. Given the formation of the Fe^2+^–buprofezin complex at the anodic sites of the CS surface, this suggests that buprofezin has coordinated with Fe^2+^ through the oxygen of the carboxyl group and the nitrogen of the amine group.^58^

#### 3.7.2 SEM/EDX tests

**Fig. 11:**
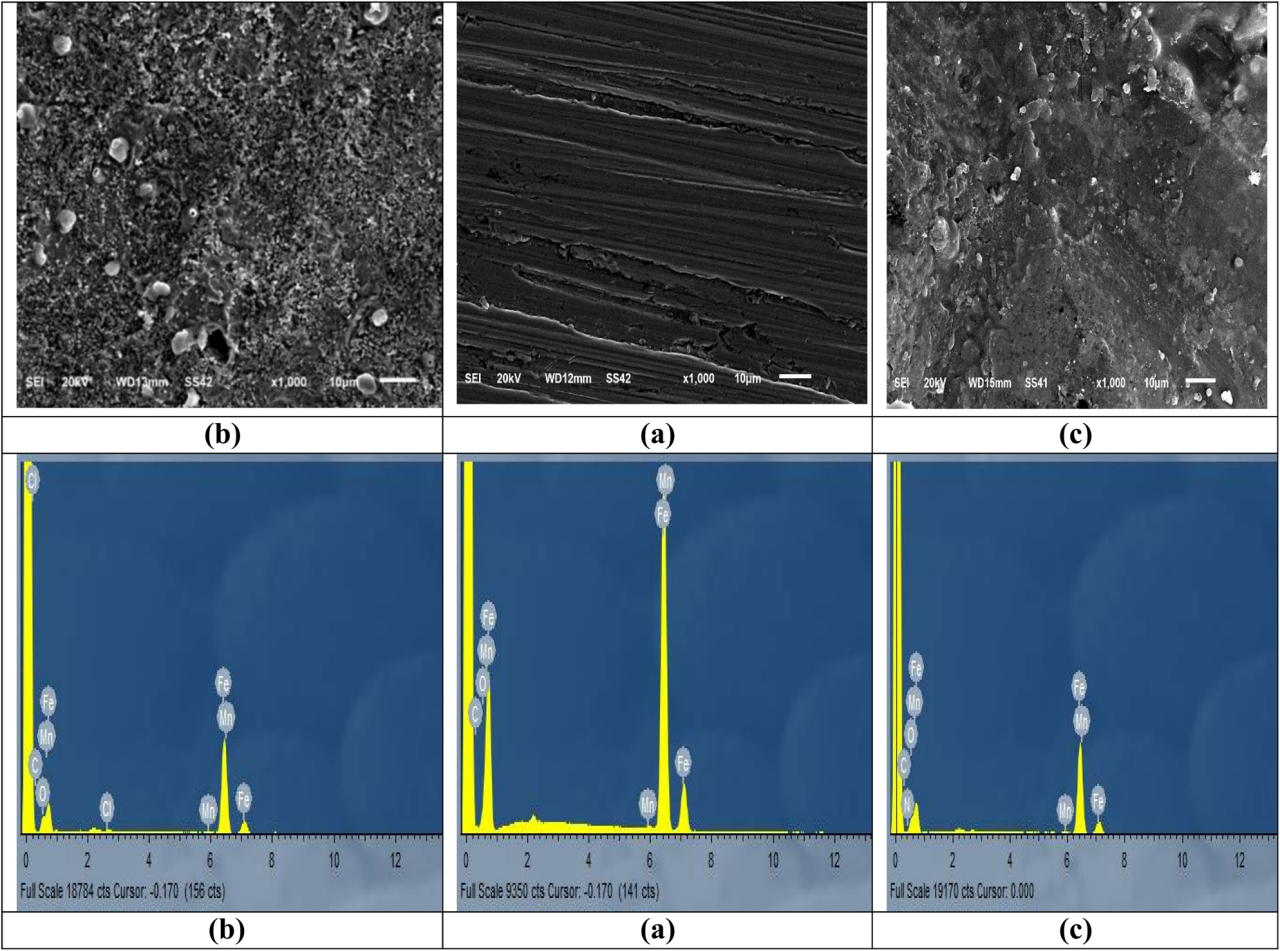
SEM and EDX image of CS surface (a) before dipping in 1 M HCl (free), (b) after 24 hours of dipping in 1 M HCl (blank) and (c) after 24 hours of dipping in 1 M HCl + 21 × 10^−6^ M of buprofezin at 25 °C.

To clarify the CS surface after exposure to an acidic solution with and without buprofezin (Fig. 11a–c), the CS surface was analyzed using SEM and EDX. The CS surface has significantly deteriorated in the blank solution due to corrosion attack (Fig. 11b). The micrograph of CS sheets in the absence of 21 × 10^−6^ M of buprofezin following a single day of dipping is displayed in Fig. 11c. By forming a shielding layer and blocking the active areas, buprofezin adsorption on the CS surface causes it to become smoother and inhibited.^59^ The elemental composition of the CS surface was determined by EDX spectroscopy both before and after a day of immersion in uninhibited and inhibited 1 M HCl (Fig. 11a–c). The EDX spectrum for free CS showed peaks that corresponded to the surface (Fig. 11a), while the spectrum for CS exposed to 1 M HCl showed peaks that corresponded to C and O (Fig. 11b and Table 6). However, the existence of adsorbed buprofezin was shown by the extra peaks corresponding to nitrogen and sulfur in the EDX spectrum of CS subjected to inhibition by 1 M HCl with 21 × 10^−6^ M concentration of buprofezin (Table 6 and Fig. 11c).

**Table 6:** Percentage atomic contents of elements obtained from EDX spectra

Samples	Wt%						
	Fe	Mn	C	Cl	O	N	S
Free CS	99.25	0.64	0.11	—	—	—	—
Blank (CS + 1 M HCl only)	83.95	0.37	3.51	0.87	11.3	—	—
(CS + 1 M HCl + 21 × 10^−6^ buprofezin)	69.94	0.27	5.34	0.12	16.12	1.95	6.26

#### 3.7.3 Atomic force microscopy (AFM) characterization

**Fig. 12:**
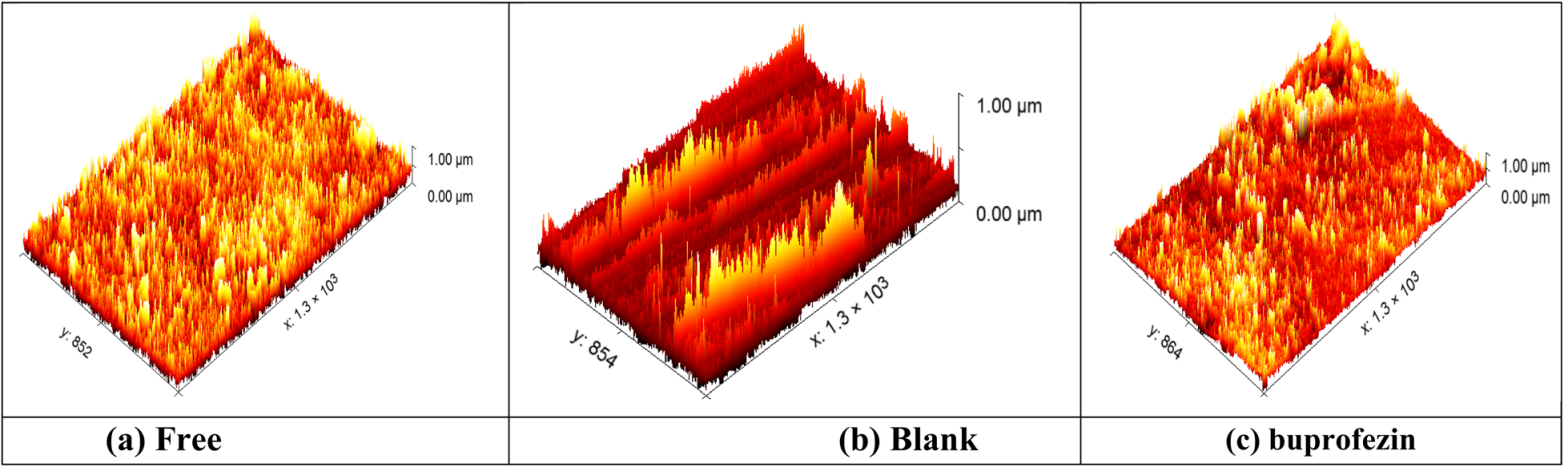
AFM 3D images of CS free (a), and in 1 M HCl (b) and with 21 × 10^−6^ M buprofezin (c) after dipping in the solution for 24 h.

AFM provides images of atomic or near-atomic-resolution surface topography, allowing Angstrom-scale determination of surface roughness.^60^ The AFM image of a polished CS surface as a standard coin (*S*_a_ = 12.45 nm) is shown in three dimensions (Fig. 12a); the CS surface after dipping in 1 M HCl as a blank sample (*S*_a_ = 689) is show in Fig. 12b; and the CS surface after dipping in 1 M HCl + 21 × 10^−6^ M of buprofezin (*S*_a_ = 124) is shown in Fig. 12c.^61^ AFM measures peak height (*S*_p_), average root mean (*S*_q_), and mean roughness (*S*_a_).

### 3.8. Theoretical studies

#### 3.8.1 Quantum calculations

**Fig. 13:**
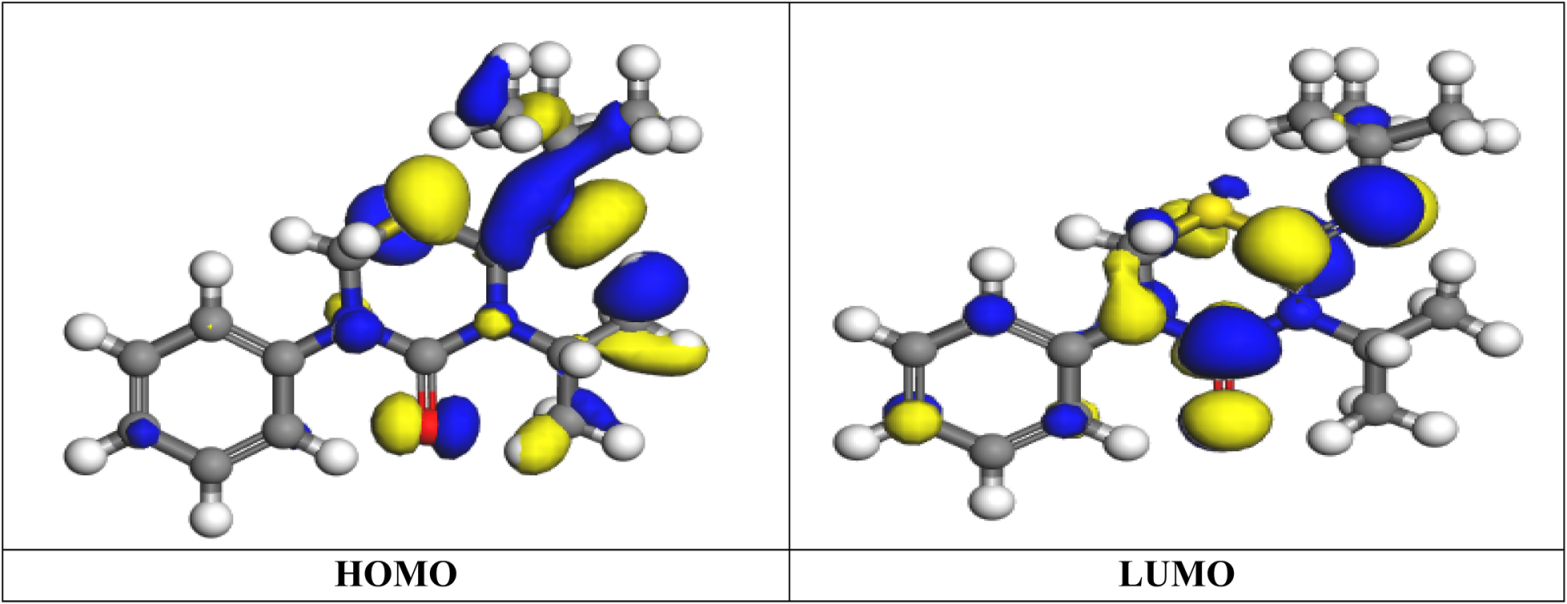
The frontier molecular orbitals provide the electron density maps of HOMO and LUMO for buprofezin.

The effect of the buprofezin inhibitor's ring structure on the productivity of the inhibition mechanism can be investigated by a few quantum computations. The geometric and electronic structure of the buprofezin inhibitor can be obtained by optimizing bond lengths and bond angles. The optimized molecular structures of the buprofezin inhibitor are shown in Fig. 13. We enlarged some data from the quantum parameter for the investigated buprofezin are as follows: as the value of Δ*E* represents the energy required to remove an electron from the highest occupied orbital, a lower Δ*E* value is generally preferred:

14 Δ*E* = *E*_LUMO_ − *E*_HOMO_

Ionization potential (*I*) and electron affinity (*A*) are related to the highest occupied molecular orbital (*E*_HOMO_) and lowest unoccupied molecular orbital (*E*_LUMO_), respectively, highlighting the connection between these properties. Absolute electronegativity (*χ*) and hardness (*η*) are calculated using specific equations. The global softness (*σ*) of the inhibitor molecule can be determined using eqn (15)–(19).

15 *I* = −*E*_HOMO_

16 *A* = −*E*_LUMO_

17
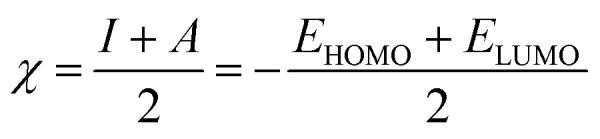
18
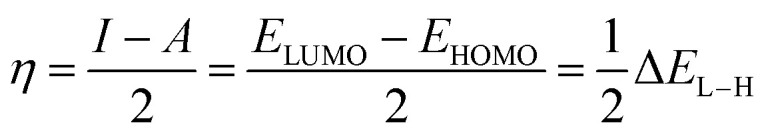
19
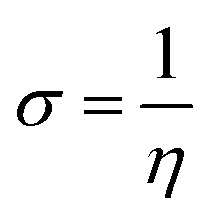


Buprofezin is more reactive and inhibits more effectively when its global softness value is lower. A greater dipole moment facilitates buprofezin's adsorption onto the CS surface. As indicated in Table 7, the inhibitor's large dipole moment is a result of the strong dipole–dipole interactions it has with the metal surface. The selectivity and reactivity of the molecule are correlated with parameters like global hardness and softness.^62^

**Table 7:** Quantum calculation parameters for buprofezin obtained from DFT

Parameter (variable)	DFT
−*E*_HOMO_ (eV)	5.233
−*E*_LUMO_ (eV)	3.886
Δ*E*, (eV), (*E*_L_ − *E*_H_)	1.35
*µ* (Debye) (dipole moment)	11.74
*A* (eV) (electron affinity)	3.89
*I* (eV) (ionization potential)	5.23
*χ* (eV) (electronegativity)	4.56
*η* (eV) (global hardness)	0.67
Δ*N*	1.81
*σ* (eV^−1^) (softness)	1.48

#### 3.8.2 Monte Carlo (MC) simulation

**Fig. 14:**
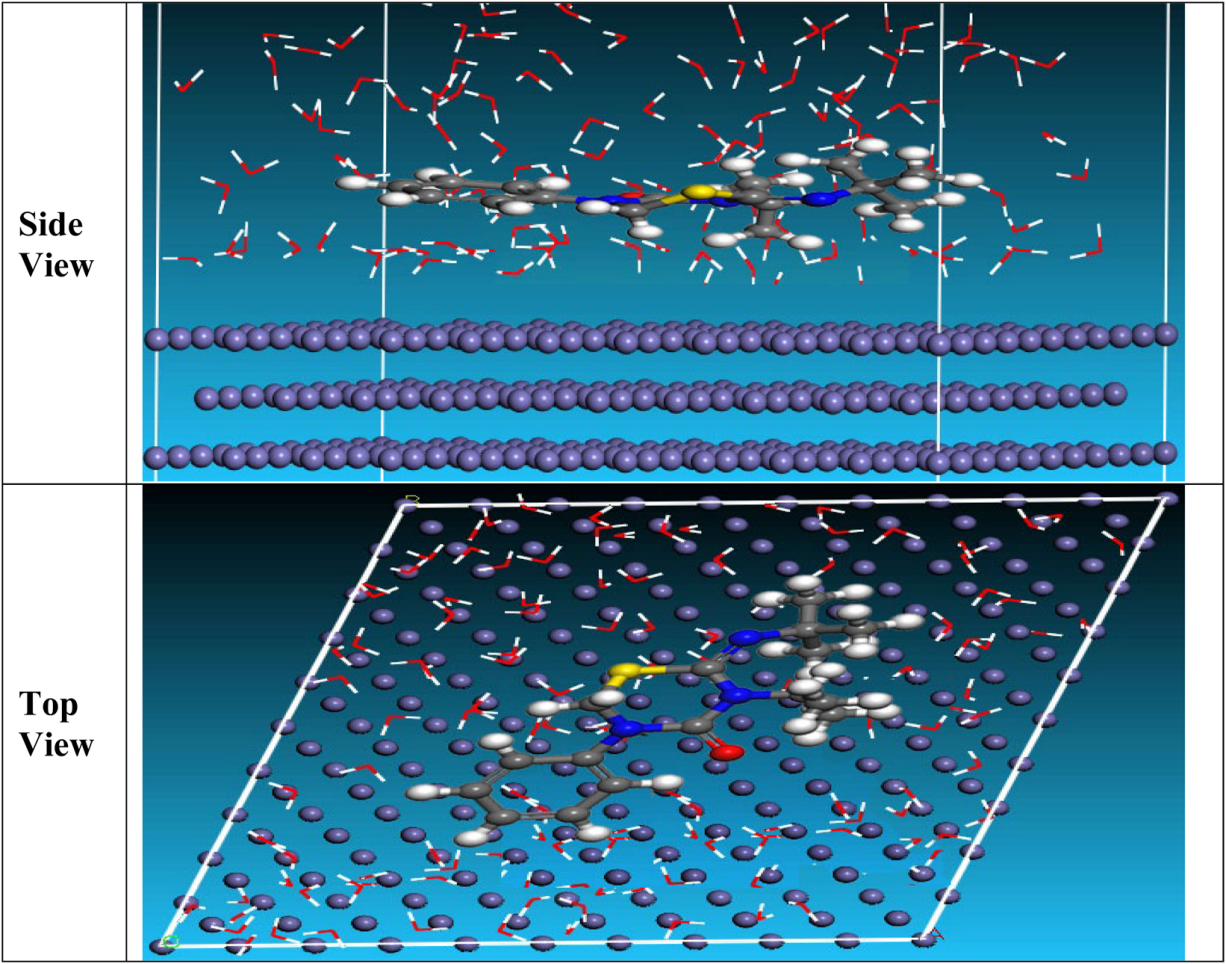
The most appropriate conformation for the adsorption of the buprofezin molecule on Fe (1 1 0).

The side and top observations of the most suitable adsorption formations for the buprofezin tested on CS surface obtained from the adsorption locator module are thus shown in Fig. 14. Adsorption energy is characterized as declining energy when materials are mixed during the adsorption process, in which an electron, ion, or molecule (adsorbent) is bound to the solid surface. As seen in Table 8, buprofezin has a higher adsorption energy, indicating strong adsorption on the hardened surface of CS and the formation of stable adsorbed layers that protect the CS from corrosion.^63,64^

**Table 8:** Results and descriptors measured by the Monte Carlo simulation for adsorption of buprofezin on iron (1 1 0)

Structure	Adsorption energy	Rigid adsorption energy	Deformation energy	Compound d*E*_ad_/dNi	H_2_O d*E*_ad_/dNi
Fe (1 1 0)/buprofezin/H_2_O	−1195.651	−1257.531	61.88	−74.31	-13.88

### 3.9. Mechanism of corrosion inhibition

**Fig. 15:**
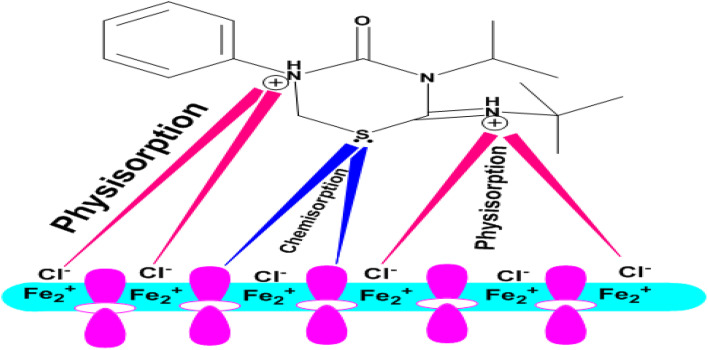
Schematic representation of the adsorption of buprofezin on CS in a 1 M HCl.

The adsorption of the inhibitor molecules on the metal surface, creating a protective barrier that stops the metal suspension, is the primary mechanism of the inhibitory processes of the produced buprofezin corrosion inhibitor. The presence of functional groups that can firmly bind the inhibitor molecules on the metal surface accounts for the inhibitor's affinity to be adsorbed on the surface. Because an inhibitor molecule forms a protective layer at the metal/solution interface, the inhibition efficacy increases as the inhibitor concentration increases. By raising the inhibitor concentration, this layer thickens and becomes more effective at preventing CS corrosion in a 1 M HCl solution. Additionally, an electrochemical study employing PDP measurements reveals that the produced inhibitor functions as a mixed-type inhibitor that inhibits both anodic and cathodic reactions.^65^ The inhibition efficacy declines with temperature, indicating that the adsorption process is physisorption. Usually, it is possible to consider two adsorption modes. Neutral buprofezin can adsorb onto the metal surface *via* chemisorption, which involves displacing water molecules from the surface and forming electron-sharing interactions between the oxygen and nitrogen atoms of buprofezin and the iron atoms of the metal. The buprofezin molecules can also be adsorbed on the metal surface due to donor–acceptor interactions between the heterocyclic π-electrons and the empty d-orbitals of iron. The protonated buprofezin in the acid medium may be adsorbed onto the metal surface, which is positively charged in acidic solution,^66^ so it is difficult for the protonated molecules to approach the positively charged CS surface (H_3_O^+^/metal surface) due to the electrostatic repulsion. The positively charged CS surface prefers Cl^−^ adsorption to create a negatively charged surface, making the adsorption of cations in solution easier.^67^ Thus, inhibition of CS corrosion in 1 M HCl is due to either the formation of metal complexes of Fe^2+^ and buprofezin derivatives or through electrostatic interactions between the positive molecules and already adsorbed chloride ions. van der Waals forces may allow these complexes to stick to the CS surface and form a protective layer (Fig. 15).

## 4. Conclusions

Buprofezin successfully prevented CS from corroding in a 1 M HCl solution. Utilizing PDP, EIS, and WL methods, the effectiveness of buprofezin as a protective film was assessed. Buprofezin demonstrated an impressive percentage IE for CS in 1 M HCl medium. While it declined at higher temperatures, the anticorrosion efficacy improved as the buprofezin concentration rose, reaching 89.7% efficiency at 21 × 10^−6^ M. The primary reason for the inhibition was buprofezin adsorption on the CS surface. The Langmuir model matched the adsorption. Thermodynamic characteristics indicated primarily physisorption but also mixed adsorption. Buprofezin is a mixed-type inhibitor. These studies are supported by the micrographs and spectra from SEM, EDX, AFM, and FTIR. In addition to the π-electrons on the benzene rings, the theoretical results showed that buprofezin was highly attached to the CS surface *via* the molecule's lone pair of electrons. −1195.651 kJ mol^−1^ was the adsorption energy between the CS surface and buprofezin. The electrochemical methods (EIS and PDP) and the WL showed good agreement. The brief immersion duration, which might not accurately represent long-term inhibitor performance, was a limitation of the study. The precise adsorption mechanism could not be confirmed due to insufficient surface characterization. Long-term immersion experiments should be included in future research to more accurately evaluate durability. Adsorption behaviour should be verified using advanced surface analysis methods (XPS, AFM, TEM).

## Conflicts of interest

The authors state that there are no conflicts of interest.

## Supplementary Material

RA-015-D5RA05962C-s001

## Data Availability

The data that support the findings of this study are available from the corresponding author, A. S. Fouda, upon reasonable request. Supplementary information (SI) is available. See DOI: https://doi.org/10.1039/d5ra05962c.
