# Supplementary material for: Buprofezin as a potent corrosion inhibitor for carbon steel in 1 M HCl solution
Source: RSC Adv. 2025 Dec 17;15(59):50652–65. doi: 10.1039/d5ra05962c (PMC12709587; doi:10.1039/d5ra05962c)
Supplement: RA-015-D5RA05962C-s001 [file RA-015-D5RA05962C-s001.pdf]

Supplementary Information (SI) for RSC Advances.
This journal is © The Royal Society of Chemistry 2025

Table S1. Inhibition was statistically compared to the efficacy of other heterocyclic organic Inhibitors that had previously been investigated

Corrosion Inhibitor	Metal	Corrosive solution	% efficiency	Ref.
(E)-N0 -(2,4-dimethoxybenzylidene)-2-(6-methoxynaphthalen-2-yl)propanehydrazide	MS	1 M HCl	95.7-95	1
N0-cyclohexylidene-2-(6-methoxynaphthalen-2-yl) propanehydrazide	MS	1 M HCl	86.0	1
Furoin thiosemicarbazone	MS	1 M HCl	89.7	2
4-pyridinecarboxaldehyde thiosemicarbazone	MS	1 M HCl	85.5	3
5-hexylsulfanyl-1,2,4-triazole	MS	1 M HCl	97.0	4
3-(3-formyl-4-hydroxy- l-phenylazo) - l, 2, 4-triazole	Cu	1 M HNO3	95.8	5
3-(3-formyl-4-hydroxy- l-phenylazo) - l, 2, 4-triazole	MS	1M HCl	92.8	6
2- , 2-amino-7-hydroxy-4-(4-methoxyphenyl)-1,4 dihydroquinoline-3 carbonitrile	MS	1 M HCl	96.6	6
2- , 2-amino-7-hydroxy-4-(4-methoxyphenyl)-1,4 dihydroquinoline-3 carbonitrile	MS	1 M HCl	98.0	6
2-amino-7-hydroxy-4-phenyl-1,4-dihydroquinoline-3-carbonitrile	MS	HCl	93.3	6
2-amino-7-hydroxy-4-(p-tolyl)-1,4 dihydroquinoline-3-carbonitrile	MS	HCl	92.8	6
5-(2- Hydroxyphenyl)-2,7-dithioxo-2,3,5,6,7,8-hexahydropyrimido [4,5-d]-pyrimidin-4(1H) one	N80 steel	1 M HCl	73.1	7
5-(2,5-dimethylthiophen-3yl)-4-(4-(6-(2,5-dimethylthiophen-3-yl)-2- hydroxypyrimidin-4-yl)phenyl)pyrimidin-2-ol	MS	H2SO4	98.3	8
5-(2,5-dimethylthiophen-3yl)-4-(4-(6-(2,5-dimethylthiophen-3-yl)-2-mercaptopyrimidin-4-yl)phenyl) pyrimidin-2-thiol	MS	H2SO4	99.3	8
5-(2,5-dimethylthiophen-3yl)-4-(4-(6-(2,5-dimethylthiophen-3-yl)-2-mercaptopyrimidin-4-yl)phenyl) pyrimidin-2-thiol	MS	H2SO4	99.3	8
2-((6-methyl-2-ketoquinoUne-3-yl)methylene) hydrazinecarbothioamide	MS	1 M HCl	95.8	9
5,50-(1,4-phenylene)bis(N-phenyl-1,3,4-thiadiazol-2-amine)	MS	1 M HCl	94.0	10
(Z)-2-tert-butylimino-3-isopropyl-5-phenyl-1,3,5-thiadiazinane-4-one (Buprofezein)	CS	1 M HCl	91.3	Our results

References

1. Chaouiki, A.; Chafiq, M.; Lgaz, H.; Al-Hadeethi, M.; Ali, I.; Masroor, S.; Chung, I. Green Corrosion Inhibition of Mild Steel by Hydrazone Derivatives in 1.0 M HCl. Coatings **2020**, 10, 640.
2. Jacob, K.; Parameswaran, G. Corrosion inhibition of mild steel in hydrochloric acid solution by Schiff base furoin thiosemicarbazone. Corros. Sci. **2010**, 52, 224–228.

3. Xu, B.; Yang, W.; Liu, Y.; Yin, X.; Gong, W.; Chen, Y. Experimental and theoretical evaluation of two pyridine carboxaldehyde thiosemicarbazone compounds as corrosion inhibitors for mild steel in hydrochloric acid solution. Corros. Sci. **2014**, 78, 260–268.
4. Naciri, M.; El Aoufir, Y.; Lgaz, H.; Lazrak, F.; Ghanimi, A.; Guenbour, A.; Ali, I.; El Moudane, M.; Taoufik, J.; Chung, J. Exploring the potential of a new 1,2,4-triazole derivative for corrosion protection of carbon steel in HCl: A computational and experimental evaluation. Colloids Surf. A Physicochem. Eng. Asp. **2020**, 597, 124604.
5. Madkour, L.; Kaya, S.; Obot, I. Computational, Monte Carlo simulation and experimental studies of some arylazotriazoles (AATR) and their copper complexes in corrosion inhibition process. J. Mol. Liq. **2018**, 260, 351–374.
6. Singh, P.; Srivastava, V.; Quraishi, M. Novel quinoline derivatives as green corrosion inhibitors for mild steel in acidic medium: Electrochemical, SEM, AFM, and XPS studies. J. Mol. Liq. **2016**, 216, 164–173.
7. Haque, J.; Ansari, K.R.; Srivastava, V.; Quraishi, M.A.; Obot, I.B. Pyrimidine derivatives as novel acidizing corrosion inhibitors for N80 steel useful for petroleum industry: A combined experimental and theoretical approach. J. Ind. Eng. Chem. **2017**, 49, 176–188.
8. Amusia, N.; Saranya, J.; Sounthari, P.; Zarrouk, A.; Chitr, S. Corrosion inhibition and adsorption behavior of some bipyridine derivatives on mild steel in acidic medium. J. Mol. Liq. **2017**, 225, 406–417.
9. Al-Baghdadi, S.B.; Al-Amiery, A.A.; Gaaz, T.S.; Kadhum, A.A.H. Terephthalohydrazide and isophthalo-hydrazide as new corrosion inhibitors for mild steel in hydrochloric acid: Experimental and theoretical approaches. Koroze Ochr. Mater. **2021**,65, 12–22.
10. Al-Amiery, A.A. Anti-corrosion performance of 2-isonicotinoyl-n-phenylhydrazine carbothioamide for mild steel hydrochloric acid solution: Insights from experimental measurements and quantum chemical calculations. Surf. Rev. Lett. **2021**, 28, 2050058.
